# Pan-Genome-Wide Investigation and Expression Analysis of *GATA* Gene Family in Maize

**DOI:** 10.3390/plants14111693

**Published:** 2025-06-01

**Authors:** Fangfang Zhao, Xia Li, Ziqi Chen, Changhong Guo

**Affiliations:** 1 Key Laboratory of Molecular Cytogenetics and Genetic Breeding of Heilongjiang Province, College of Life Science and Technology, Harbin Normal University, Harbin 150025, China; lnilyf@163.com (F.Z.); lx627@126.com (X.L.); 2 Jilin Provincial Key Laboratory of Agricultural Biotechnology, Institute of Agricultural Biotechnology, Jilin Academy of Agricultural Sciences (Northeast Innovation Center of Agricultural Science and Technology in China), Changchun 130124, China

**Keywords:** GATA, pan-genome, structural variation, positive selection, abiotic stress

## Abstract

GATA is a crucial transcription factor involved in plant growth, development, and responses to abiotic stress. Therefore, identifying and exploring GATA transcription factors in maize is of significant importance. In this study, we identified 75 *ZmGATA* genes based on the pan-genome of maize, which includes 26 high-quality maize genomes. These consist of 58 core genes (present in all 26 lines), 12 non-essential genes (present in 2 to 23 lines), 2 near-core genes (present in 24 to 25 lines), and 3 private genes (present in only 1 line). By evaluating the Ka/Ks ratio of the *ZmGATA* genes in 26 maize varieties, we found that the Ka/Ks ratios of *ZmGATA31*, *ZmGATA32*, *ZmGATA36*, and *ZmGATA9* were greater than 1, which may indicate that these four genes are under positive selection. In contrast, the Ka/Ks ratios of other *ZmGATA* genes were less than 1, suggesting that these genes may be under purifying selection. In the 26 maize genomes, we observed a significant difference in the expression of *ZmGATA8* between varieties affected by structural variations (SVs) and those not affected. In certain varieties, SVs altered conserved structures. Additionally, we analyzed the expression levels of *ZmGATA* genes in different maize tissues and under abiotic stress. *ZmGATA38* and *ZmGATA39* were highly expressed in the endosperm, thereby influencing starch synthesis, while *ZmGATA7*, *ZmGATA10*, *ZmGATA19*, *ZmGATA28*, and *ZmGATA40* were found to be associated with abiotic stress responses. These findings provide valuable new resources for functional research on *ZmGATA*.

## 1. Introduction

Plant transcription factors are a class of proteins that have DNA-binding domains and can regulate gene expression. They can regulate RNA polymerase binding and the activation or inhibition of gene transcription by binding to specific DNA sequences [1]. Common transcription factors in plant species include bHLH, bZIP, NAC, WRKY, MYB, and GATA. They can participate in regulating multiple biological processes in plants, such as growth and development, secondary metabolite synthesis, disease resistance, and stress responses [2,3,4,5,6,7].

GATA factors were first identified as proteins that interact with the conserved WGATAR (W=T or A; R=G or A) involved in vertebrate red-lineage-specific gene expression motifs [8]. The GATA factor is characterized by the presence of a conserved type IV zinc finger motif [9]. GATA proteins can regulate gene expression by recognizing and binding to cis-acting elements (T/A) GATA (A/G) in the promoter regions of genes. Animal factors typically contain two C-X2-C-X17-C-X2-C zinc finger structural domains [10]. Most known fungal GATA factors contain a single C-X2-C-X17-C-X2-C finger, very similar to the carboxyl (C)-terminated finger of the animal GATA factor. Several examples of fungal GATA factors containing variant C-X2-C-X18-C-X2-C DNA-binding structural domains are also known [11]. Examples of C-X2-C-X17-CX2-C (type IV a) and C-X2-C-X18-C-X2-C (type IV b) GATA factors have been found in fungi [12]; animals contain only the former structure, and plants contain only the latter. Plant GATA transcription factors usually contain a single zinc finger structural domain with amino acid residues 18 (C-X2-C-X18-C-X2-C) or 20 (C-X2-C-X20-C-X2-C) [13]. In the 1990s, scientists identified GATA motifs in the regulatory regions of certain light-responsive genes and discovered that GATA protein factors could bind these GATA elements [14]. However, the tobacco *NtGATA1* gene was the first GATA transcription factor cloned in a plant gene [15]. With the release of more information on plant genomes, *GATA* family genes have been successfully identified in many species, such as *Oryza sativa* [16], *Arabidopsis thaliana* [12], *Glycine max* [17], and *Ophiorrhiza pumila* [18], *Phyllostachys edulis* [19], *Brachypodium distachyon* [20], *Capsicum annuum* [21], *Cucumis sativus* [22], *Gossypium genus* [23], and so on. The *GATA* genes of plants are divided into subfamilies I, II, III, and IV based on motif species, conserved sequences, and evolutionary relationships [22].

GATA transcription factors are reported to be extensively involved in plant growth and development. They serve as important regulators of multiple biological processes, including nitrogen metabolism, stress response, flowering, and hormone signaling. *PdGATA19* in poplar regulates secondary xylem differentiation, and the overexpression of this gene increased biomass accumulation, and chlorophyll content by 20%, and enhanced photosynthetic efficiency [24]. The DELLA protein, a negative regulator of gibberellin, inhibits the function of transcription factors known as PIFs. These PIFs are capable of binding to the promoter of the *Arabidopsis* GATA transcription factor GNL, thereby influencing seed germination [25,26]. Additionally, the transcription factor BME3 (*AtGATA8*) is present in high levels within seeds, which exhibit profound dormancy following its knockdown. However, germination can be restored through the external application of GA3 [27]. Early discoveries related to the function of plant GATA in nitrate metabolism were driven by the discovery that several fungal *GATAs* regulate nitrogen responses and that GATA transcription factors play a role in nitrogen metabolite repression, controlling the transcription of genes related to nitrogen uptake and catabolism [28]. *Arabidopsis* GATA transcription factors *ZML1* and *ZML2* correspond to mutants zml1 and zml2 with significantly slower cotyledon yellowing than the wild type, implying that *ZML2* and *ZML1* are essential components of the Cry1-mediated photoprotective response pathway [29,30]. The expression of soybean *GmGATA44* and *GmGATA58* was reduced by 81% and 79% under low-nitrogen stress, indicating that *GATA* transcription factors are sensitive to nitrogen stress [17].

As a vital dual-purpose crop supporting global food security and economic systems, maize (*Zea mays*) requires optimized yield and nutritional quality. These key determinants are significantly compromised by abiotic stressors (drought, salinity, temperature extremes), with starch metabolism emerging as a central regulatory hub that coordinates carbohydrate allocation between yield formation and grain quality parameters [31]. Pan-gene family analysis can identify more members than a single gene family, which is beneficial for studying key genes across different species. Currently, this method has been widely used in crops such as rice [32] and barley [7]. In this study, we present a systematic pan-genome analysis of the GATA transcription factor family. Using an integrative approach that combines comparative genomics, phylogenetics, and functional annotation, we aim to elucidate the evolutionary dynamics and functional diversity of *GATA* genes. Our analysis encompasses multiple species from diverse lineages, providing insights into the conserved and lineage-specific roles of GATA TFs. Furthermore, we explore the potential regulatory networks and biological processes mediated by GATA TFs, highlighting their significance in both fundamental biology and applied research fields. This research contributes to a deeper understanding of the evolution and functional complexity of the GATA transcription factor family, offering a valuable resource for future studies in gene regulation and evolutionary genomics.

## 2. Results

### 2.1. Pan-Genome-Wide ZmGATA Identification

**Figure 1:**
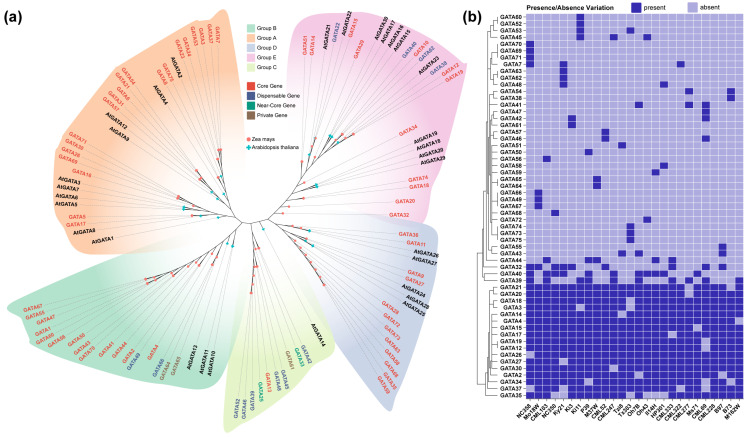
The evolution and presence/absence analysis of maize GATA proteins. (**a**) Phylogenetic tree of maize and *Arabidopsis GATA* genes. In the phylogenetic tree, different groups are filled with different colors: orange for Group A, light green for Group B, yellow for Group C, purple for Group D, and pink for Group E. Different types of genes are represented by different colors: red for core genes, blue for dispensable genes, green for near-core genes, and brown for private genes. Different shapes on the branches represent different species: a cross for *Arabidopsis* and a circle for maize. (**b**) Heatmap of the presence and absence of *GATAs* in 26 maize varieties. Dark purple indicates the presence of a gene, while light purple indicates the absence of a gene.

This study identified 75 *ZmGATA* genes in the maize genome, including 58 core genes, 12 non-essential genes, 2 near-core genes, and 3 private genes. In the reference genome, 41 *GATA* genes were identified, of which 6 contain two typical GATA domains, while 35 contain one GATA domain (Figure S1). A phylogenetic tree was constructed using *ZmGATA* protein sequences provided by Hufford et al. [33]. Since the grouping of *Arabidopsis* GATA proteins has been previously reported, we classified the maize GATA proteins based on this grouping. Based on the classification of *AtGATA*, we divided *ZmGATA* (Figure 1a) into five subgroups. Group A, the largest subgroup, contains 20 *GATA* genes, followed by Group B with 17 genes, Group E with 16 genes, and Group D with 12 genes. Group C is the smallest subgroup, with only 10 *GATA* genes. All genes in Groups A and D are core genes. Figure 1b demonstrates the presence or absence of *ZmGATAs* in 26 inbred maize lines. Some genes exhibit a variety-specific presence, such as *GATA60* and *GATA52*, which are found only in Ki11; *GATA61* and *GATA64/GATA65* are found only in Ki3 and M37W, respectively. These findings suggest that these genes may be associated with traits unique to these varieties.

### 2.2. ZmGATA Is Subjected to Different Selection Pressures Among Maize Varieties

**Figure 2:**
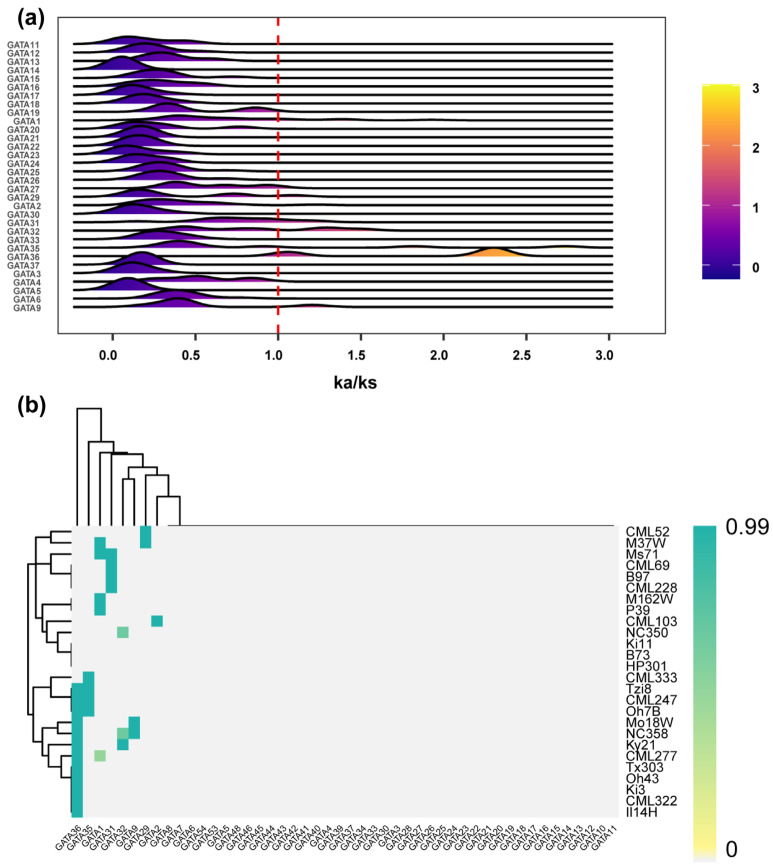
Ka/Ks values of *ZmGATA*. (**a**) Distribution of Ka/Ks values of *ZmGATA* in 26 maize varieties. The x-axis represents the range of the Ka/Ks values, while the height and color of the peaks in the figure indicate the number of gene accumulations in different maize varieties. (**b**) Heatmap of the frequency of occurrence of different maize varieties for each *GATA* with a Ka/Ks ratio > 1. The x-axis represents maize *GATA* genes, and the y-axis represents different maize varieties. The colored blocks in the figure indicate the ratio of *GATA* genes with a Ka/Ks value greater than 1 in different varieties. Among them, *GATA36*, *GATA35*, *GATA1*, *GATA31*, *GATA32*, *GATA9*, *GATA29*, and *GATA2* have a higher ratio of Ka/Ks values greater than 1 in various maize varieties.

Ka/Ks ratio analysis provides valuable insights into the selection pressures acting on gene family members during the evolution of different varieties. To explore the selection pressures on *ZmGATA* genes, we calculated the Ka/Ks ratio for each *GATA* gene (Figure 2a) based on the gene sequences from 26 maize genomes. Figure 2a shows the Ks/Ks values of *GATA* in the 26 maize varieties. All Ka/Ks values for the *GATA* genes peak between 0 and 1, though the peak positions vary, with *GATA37* exhibiting a notably higher Ka/Ks value. *GATA36* shows evidence of positive selection in certain varieties, with a Ka/Ks value of between 2 and 2.5. Among the 26 maize genomes, a substantial proportion of the Ka/Ks values for *GATA31*, *GATA32*, *GATA36*, and *GATA9* exceed 1 (Figure 2b), suggesting these genes have undergone selection pressure during maize domestication. In contrast, the Ka/Ks values for the other genes are mostly greater than 0 but less than 1, indicating that these genes have been subject to purifying selection during domestication.

### 2.3. Expression and Structure of ZmGATA8 Genes Are Affected by SVs

**Figure 3:**
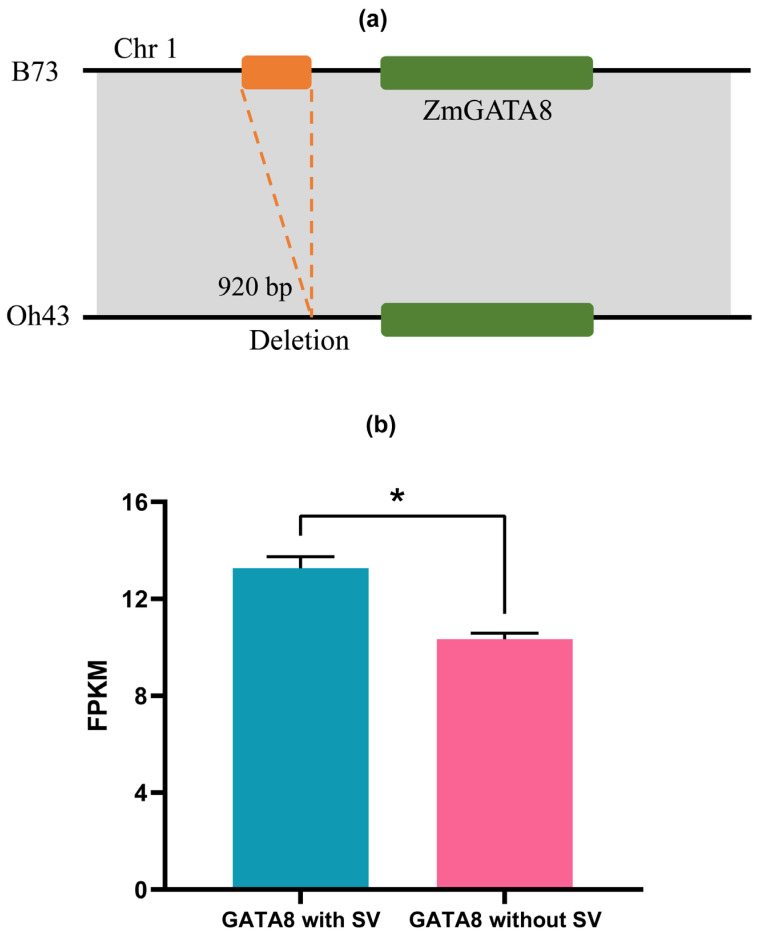
The effect of SVs on genes. (**a**) The effects of SV insertion and deletion on *ZmGATA8*. Compared to B73, the *ZmGATA8* on chromosome 1 in Oh43 exhibits a 920 bp fragment deletion upstream of the gene. (**b**) The expression of *GATA8* was significantly affected by SVs. The expression levels of the maize *GATA8* with and without the fragment deletion were calculated, and a significant difference in the expression levels was observed. * *p* < 0.05 indicates a significant difference.

A total of 181 SVs were found to overlap with the 41 *ZmGATA* gene regions and their 2 kb upstream and downstream regions. Compared to the reference genome, these structural variations (SVs) are characterized by deletions, insertions, and inversions (Figure 3a). We calculated the Pearson correlation coefficients between the expression levels of genes that overlap with SVs and those that do not. The results showed that only *ZmGATA8* exhibited a significant difference between varieties with SVs and those without SVs (*p* < 0.05 and |r| > 0.3). This suggests that SVs significantly alter the expression of *ZmGATA8* (Figure 3b). There is significant variation in *ZmGATA8* between Oh43 and B73, which may be due to their origins from different regions.

**Figure 4:**
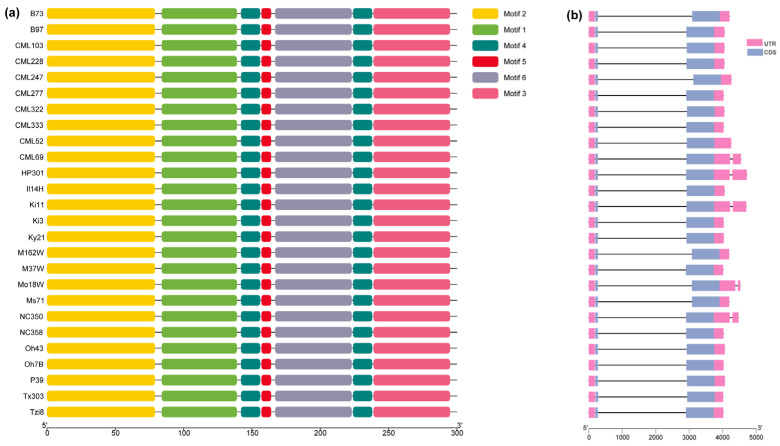
Motif count (**a**) and gene structure. By using MEME Suite to search for motifs in the *ZmGATA8* gene, six motifs were found to be conserved across different maize varieties. (**b**) The structure of *ZmGATA8* across 26 different maize varieties. The structural differences in the gene occur in the 3′ untranslated region. *ZmGATA8* has an additional 3′ untranslated region in the maize varieties CML69, HP301, Ki11, Mo18W, and NC350.

To explore whether SVs affect the gene structure of *GATA8*, we analyzed the gene structure of *ZmGATA8* across the 26 maize genomes (Figure 4). In most maize varieties, the domain types and numbers, exons, and 5′ untranslated regions of *ZmGATA8* were consistent with the reference genome (B73). However, CML69, HP301, Ki11, Mo18W, and NC350 exhibited differences in the number of units in the 3′ untranslated region, with one additional unit compared to B73.

### 2.4. Collinearity Analysis of GATA Family Genes in Maize and Other Plants

Synteny analysis is an effective method to explore the functional and evolutionary relationships between different species’ genes. To examine the evolutionary relationship between maize and other plants with *GATA* genes, an examination of the collinearity of *GATAs* was conducted.

The *GATA* genes on different chromosomes of corn are relatively conserved in grass crops. For example, there are 41, 40, 48, and 40 pairs of collinear genes in *Setaria viridis*, rice, sorghum, and foxtail millet, respectively. It is worth mentioning that there are 91 pairs of collinear genes in wheat. These collinear genes of *GATA* in these crops correspond to similar positions on the corn chromosomes. The above results indicate that the *GATA* family genes are relatively conserved in grass crops of the same family because they are all diploid organisms. Wheat, however, is an allohexaploid, and the doubling during its evolutionary process has resulted in more collinear genes of *GATA* in wheat compared to other crops.

**Figure 5:**
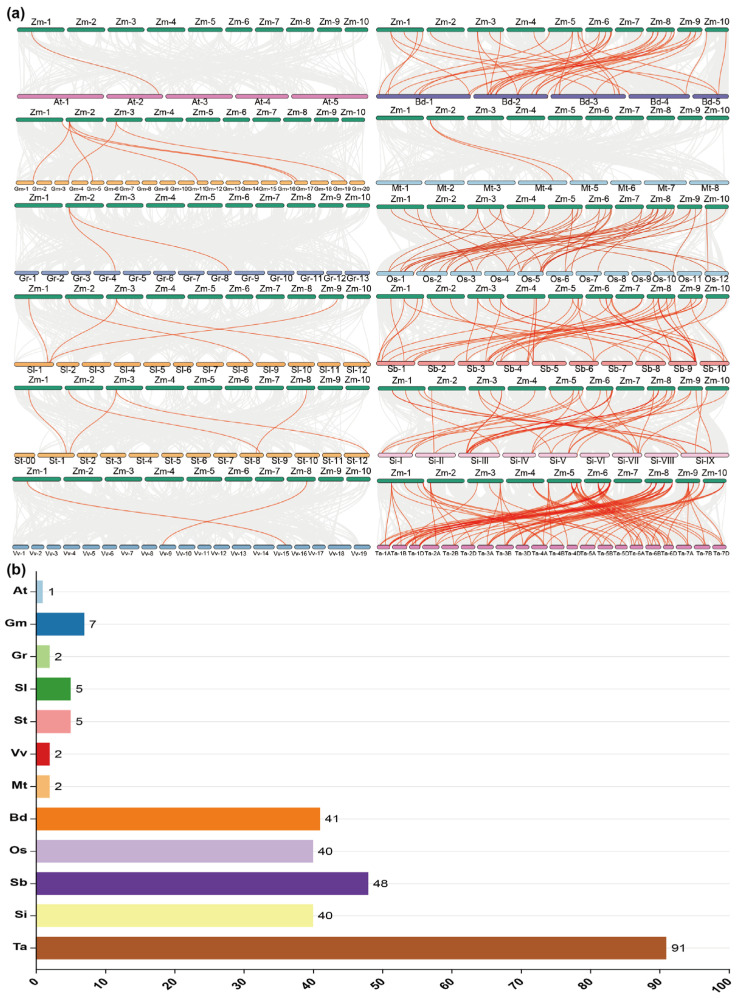
Collinearity analysis of *GATA* genes in maize and other plants. (**a**) Collinearity analysis of *GATA* genes in *Zea mays*, *Arabidopsis thaliana*, *Glycine max*, *Solanum lycopersicum*, *Solanum tuberosum*, *Vitis vinifera*, *Brachypodium distachyon*, *Medicago truncatula*, *Oryza sativa*, *Sorghum bicolor*, *Setaria italica,* and *Triticum aestivum*. The gray background lines represent all the collinearity modules, and the red lines represent gene pairs with collinearity. (**b**) The number of pairs of *GATA* collinearity genes in maize and other plants. The different colors of columns represent the number of gene pairs.

To further explore the collinearity relationship between the GATA gene in maize and other plants (Figure 5a,b), seven common plants, including *Arabidopsis thaliana* (Brassicaceae), alfalfa and soybean (Fabaceae), cotton (Malvaceae), tomato and potato (Solanaceae), and grape (Vitaceae), were selected for collinearity analysis with maize. They share one, two, seven, two, five, five, and two pairs of homologous *GATA* genes with maize, respectively. It is worth mentioning that the gene positions of the *GATA* collinear genes in maize and related plant species are similar. For example, potato and tomato correspond to the chromosomes Zm-1, Zm-2, and Zm-3 in maize, while *Medicago alfalfa* and soybean correspond to Zm-1. Therefore, *GATA* family genes have a distant evolutionary relationship with maize and these plants.

### 2.5. Three-Dimensional Structure and Interaction Analysis of Maize GATA Proteins

**Figure 6:**
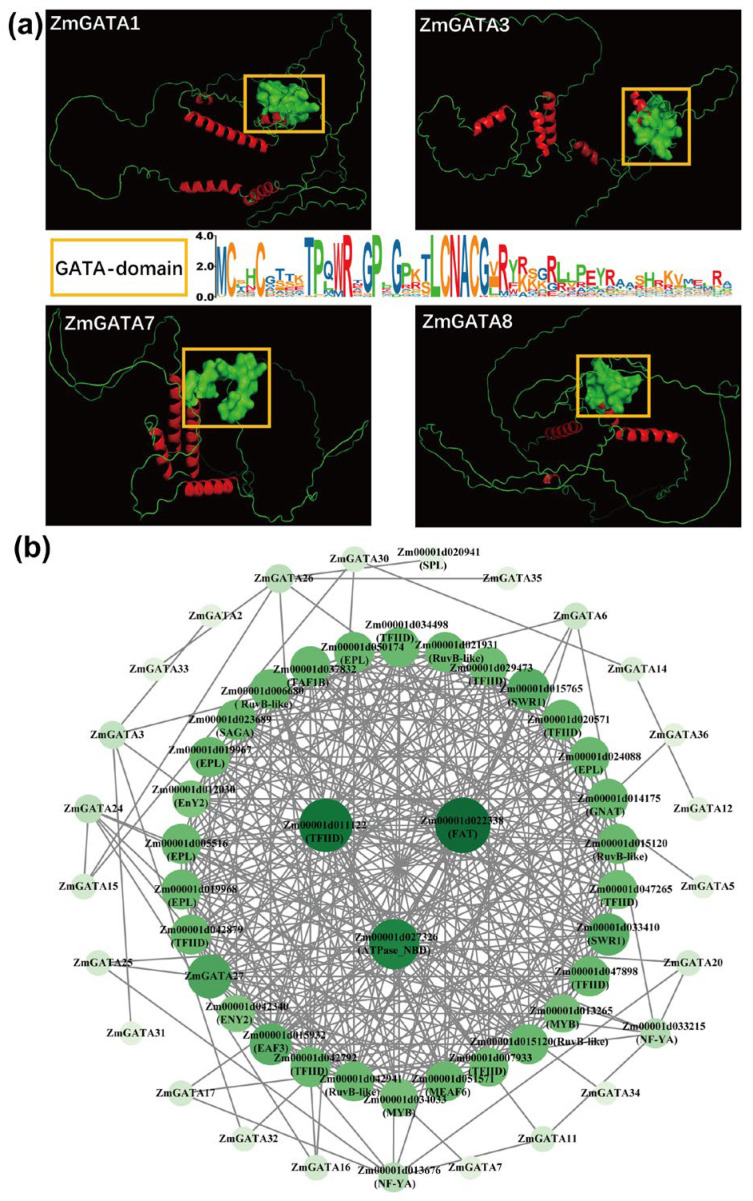
Three-dimensional structure analysis of different subfamily maize GATA proteins and protein interaction prediction analysis. (**a**) Three-dimensional structures and conserved sequences of four representative GATA proteins from different subfamilies. The yellow boxes indicate the conserved structural domains of GATA proteins indicated in ZmGATA1,ZmGATA3,ZmGATA7 and ZmGATA8. (**b**) Protein interaction network of maize GATA proteins.

The structure and interactions of proteins are crucial for their functionality. We used the STRING database to perform the interaction analysis of maize GATA proteins. After organizing the results, we calculated the interaction weight values and subsequently visualized the data using Cytoscape 3.9.1. In this study, ZmGATA1, ZmGATA3, ZmGATA7, and ZmGATA8 were selected for three-dimensional structural simulations. As shown in Figure 6a, all four proteins consist of a single polypeptide chain, each containing a different number of α-helices and a GATA structural domain module (yellow rectangle). The number of α-helices varies: ZmGATA1 and ZmGATA3 each contain five, while ZmGATA7 and ZmGATA8 have four. Additionally, the three-dimensional structures of the GATA domain differ among the proteins. ZmGATA7 is particularly distinct, as it contains a cavity, which is not observed in the other GATA proteins. The protein interaction network, shown in Figure 6b, includes several interacting protein families, such as ATPase_NBD, FAT, RuvB-like, TF Ⅱ D, SWR1, EPL, GNAT, MYB, and NF-YA. Interaction degree analysis reveals that ZmGATA27 occupies a more central position within the network, interacting with a larger number of proteins compared to other maize GATA proteins. ZmGATA7 specifically interacts with the key transcription factors NF-YA and MYB, which may be linked to its unique three-dimensional structure. These findings indicate that there are structural differences among maize GATA proteins in different subclasses, and their interactions with a variety of protein families suggest a broad functional diversity.

### 2.6. GATA Family Gene Expression Analysis in Different Tissues of Maize and Under Abiotic Stress

**Figure 7:**
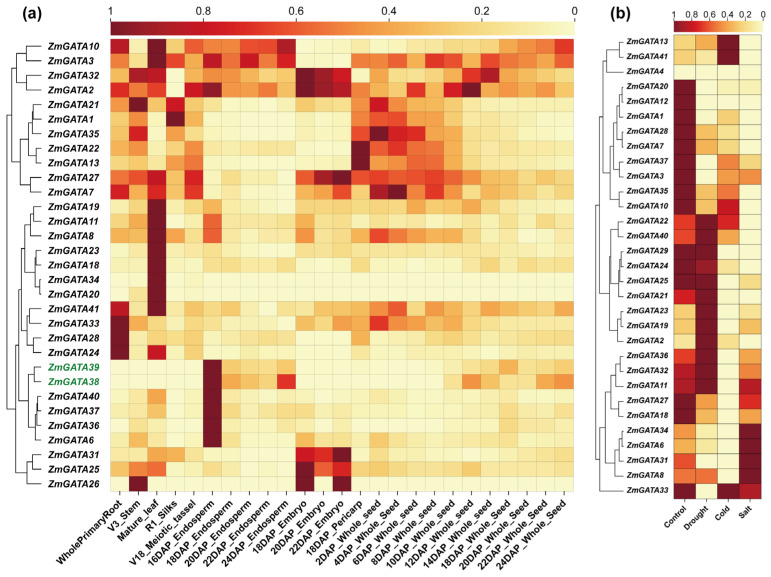
The *GATA* genes’ expression levels vary across different organs and under various abiotic stresses in maize. (**a**) The expression profiles of *GATA* genes in the roots, stems, leaves, stamens, pistils, seed coats, embryos, endosperms, and seeds at different developmental stages of corn. The ID of green genes represents genes that are specifically expressed in the endosperm and seed; DAPs: days after pollination. (**b**) The expression profile of the *GATA* genes under salt, drought, and cold stress.

The gene expression levels in different organs at different developmental stages are closely related to their functions. To further understand the function of *ZmGATAs*, the RNA-seq expression patterns of *ZmGATAs* were quantified based on FPKM values in nine different organs (Figure 7a), including roots, stems, leaves, female inflorescences, male inflorescences, pericarp, embryos (18–22 DAPs), endosperm (16–24 DAPs), and seeds (2–24 DAPs). The expression clustering analysis results showed that some genes exhibited specific expression patterns. *ZmGATA26*, *ZmGATA25*, and *ZmGATA31* were highly expressed in embryos. *ZmGATA6*, *ZmGATA36*, *ZmGATA37*, and *ZmGATA40* were highly expressed in the endosperm 16 DAPs. *ZmGATA38* and *ZmGATA39* exhibited specific expression in the endosperm 16–24 DAPs. *ZmGATA1*, *ZmGATA13*, *ZmGATA21*, *ZmGATA22*, and *ZmGATA35* showed high expression in organs other than embryos, the endosperm, and seeds 18–24 DAPs. *ZmGATA8*, *ZmGATA11*, *ZmGATA18*, *ZmGATA19*, *ZmGATA20*, *ZmGATA23*, and *ZmGATA34* exhibited specific expression in mature leaves. *ZmGATA3* was not expressed in the endosperm. These results indicate that *GATA* family genes have distinct expression patterns in different organs at different stages of maize development and exhibit differences among different subfamilies. For example, members of Subfamily I are not expressed in embryos, the endosperm, and grains before the milking stage, suggesting that they may not be involved in the physiological processes of grain filling but rather play a role in vegetative growth. Of particular note, *ZmGATA38* and *ZmGATA39* are specifically highly expressed in the endosperm and grains at the late milky stage, which may suggest their involvement in the synthesis of maize starch.

The expression levels of the maize *GATA* gene family under abiotic stress (drought, cold, and salt) are shown in Figure 7b. According to the clustering analysis results and the control comparison, the expression levels of *ZmGATA13* and *ZmGATA41* increase under low-temperature stress. The expression levels of *ZmGATA1*, *ZmGATA3*, *ZmGATA7*, *ZmGATA12*, *ZmGATA20*, *ZmGATA28*, *ZmGATA35*, and *ZmGATA37* are downregulated under drought, low-temperature, and salt stress. The expression levels of *ZmGATA2*, *ZmGATA19*, and *ZmGATA23* only increase under drought stress. Therefore, the maize *GATA* gene family may be involved in the abiotic stress response.

### 2.7. Co-Expression Network and Enrichment Analysis of Maize GATA Family Genes

**Figure 8:**
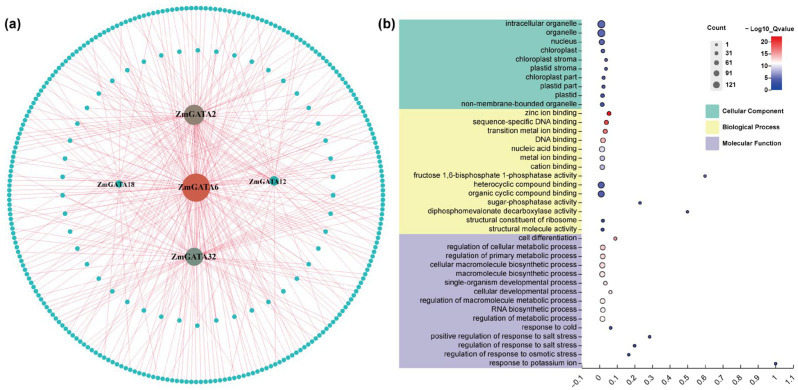
Co-expression analysis of *ZmGATAs*. (**a**) Co-expression network of *ZmGATAs*. The correlation between genes is indicated by differently sized circles. Red lines indicate the interaction links between node *ZmGATAs* and other genes. (**b**) Gene Ontology (GO) annotation of co-expressed *ZmGATA* genes.

The construction of co-expression networks and the study of gene function are closely related. We used the online platform MaizeNetome, constructed by Huazhong Agricultural University, to perform the co-expression analysis of maize *GATA* genes. Subsequently, we calculated and visualized the co-expression network using Cytoscape 3.9.1. The co-expression genes of the maize *GATA* gene family were analyzed using publicly available data based on the co-expression correlation coefficient. As shown in Figure 8a, *ZmGATA6* occupies the most central position in the entire co-expression network, followed by *ZmGATA2*, *ZmGATA32*, *ZmGATA12*, and *ZmGATA18*. To investigate the function of these genes, enrichment analysis was performed on the genes in the co-expression network. The results are shown in Figure 8b. In terms of cellular components, most of the genes are enriched in cell organelles and chloroplasts. Some genes are enriched in biological processes such as metal transport, fructose-1,6-bisphosphatase activity, and sugar-phosphorylating enzymes. In terms of molecular functions, some non-biotic stresses are enriched, such as salt, cold, and osmotic stress. These results indicate that some maize *GATA* genes can form co-expression networks with other genes and may have similar functions to them.

### 2.8. Analysis of the Correlation Between Maize GATA Family Genes and Starch Synthesis

**Figure 9:**
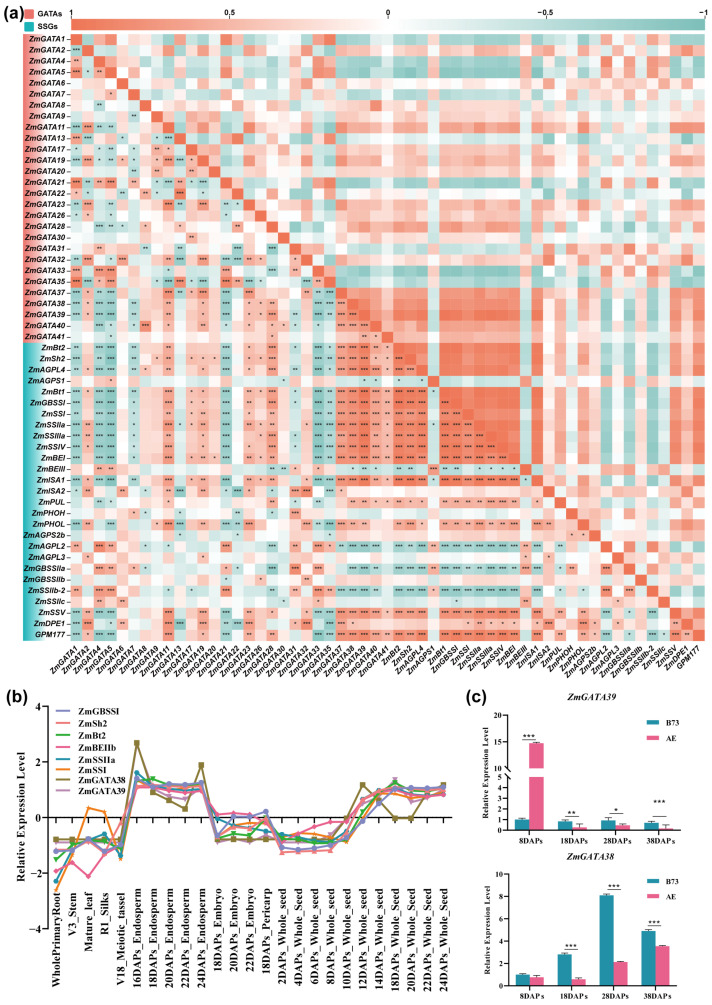
Analysis and validation of the correlation between maize GATA transcription factors and starch synthesis. (**a**) Correlation analysis of the expression levels of maize GATA transcription factors and starch-synthesis-related genes (SSGs). *ZmGATAs* and *SSGs* gene IDs are represented by pink and blue backgrounds, respectively. The correlation coefficients of gene expression levels are represented by orange and green blocks, with orange indicating a positive correlation and green indicating a negative correlation. * *p* < 0.05, ** *p* < 0.01, and *** *p* < 0.001 represent the significance of correlation coefficients. (**b**) Analysis of the tissue-specific expression patterns of maize GATA transcription factors *ZmGATA38* and *ZmGATA39* and representative genes involved in starch synthesis. Different-colored lines represent different genes, and the selected tissues include maize roots, stems, leaves, female inflorescences, and male inflorescences, as well as embryos, endosperm, and seeds at different developmental stages. (**c**) RT-qPCR analysis of the expression levels of *ZmGATA38* and *ZmGATA39* in maize with different starch contents. B73 and AE refer to maize with high and low amylose starch, respectively. The terms 8 DAPs, 18 DAPs, 28 DAPs, and 38 DAPs represent different days after maize pollination. The error bars represent the standard deviation (SD) from three biological replicates (* *p* < 0.05, ** *p* < 0.01, *** *p* < 0.001, Student’s *t*-test).

Starch is the main component of corn kernels, and a series of enzymes, including AGPases, SSs, SBEs, and DBEs, are involved in controlling starch synthesis. The genes encoding these enzymes have common expression patterns, being specifically or highly expressed in the endosperm and seeds. Transcription factors also participate in regulating starch synthesis. To investigate whether maize *GATA* transcription factors regulate starch synthesis, the co-expression correlation analysis of *ZmGATAs* and *ZmSSGs* was conducted. As shown in Figure 9a, *ZmGATA38* and *ZmGATA39* were significantly correlated with starch-synthesis-related genes, such as *ZmGBSSⅠ*, *ZmSSⅠ*, *ZmSSⅡa*, *ZmSSⅢa*, and *ZmSSⅣ*. Genes with similar expression trends in different organs at different stages may have similar functions. Thus, the expression patterns of *ZmGATA38* and *ZmGATA39* and starch-synthesis-related genes (*ZmGBSSⅠ*, *ZmSh2*, *ZmBt2*, *ZmBEⅡb*, *ZmSSⅡa*, and *ZmSSⅠ*) were analyzed, as shown in Figure 9b. We selected two corn materials, B73 and AE1, with different starch contents. Their starch content, starch branching enzyme activity, and starch content at various developmental stages of the grains are shown in Figure S3. The expression levels were downregulated in the roots, stems, leaves, seed coat, female inflorescences, male inflorescences, and embryos but upregulated in the endosperm at 16–24 days after pollination (DAPs) and in the seeds at 14–24 DAPs, indicating that *ZmGATA38* and *ZmGATA39* may be involved in maize starch synthesis. To validate this hypothesis, two maize materials with high and low total starch contents, B73 and AE1, were selected, and expression level detection was performed (Figure 9c). The expression level of *ZmGATA38* in B73, which has a high total starch content, was higher at 8 DAPs, 18 DAPs, 28 DAPs, and 38 DAPs compared to AE1, which has a low total starch content. As for *ZmGATA39*, a difference was observed at 8 DAPs, with its expression level in B73 being lower than in AE1, while the expression patterns at other time points were consistent with *ZmGATA38*. Therefore, *ZmGATA38* and *ZmGATA39* are involved in starch synthesis at different stages.

### 2.9. Quantification of Maize GATA Gene Expression Under Low-Temperature, Drought, and Salt Stress Using RT-qPCR

**Figure 10:**
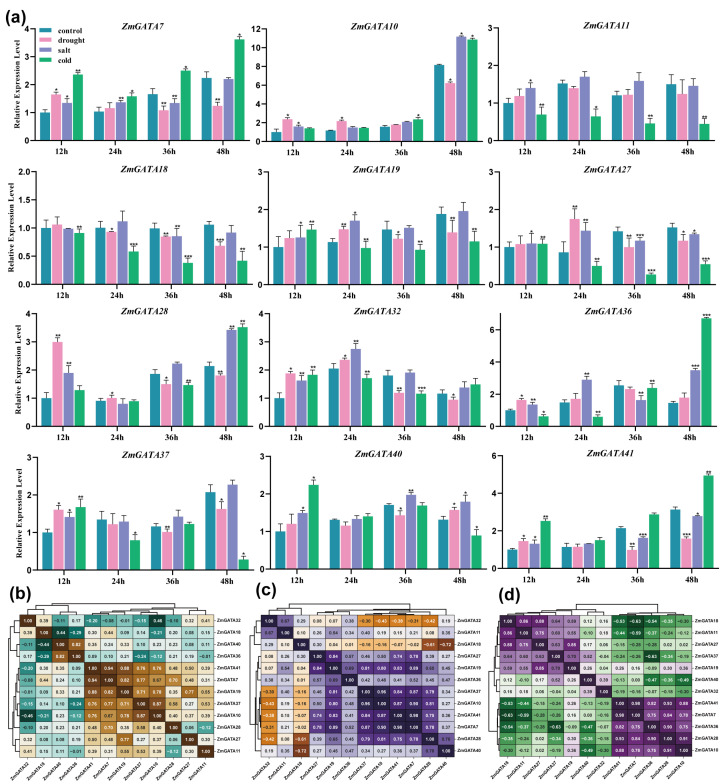
Hierarchical clustering analysis of the expression levels and correlations of eight maize *GATA* genes under different abiotic stress treatments (cold, drought, and salt) at different stress durations in seedlings. (**a**) The expression levels of *ZmGATA* genes in B73 maize seedling leaves before and after 12 h, 24 h, 36 h, and 48 h of drought, salt, and cold stress treatment simulated by 20% PEG 6000, 200 mM NaCl, and 4 °C. The data were normalized to the 18sRNA gene, and the vertical bars indicate the standard deviation. The asterisks indicate the corresponding gene significantly up- or downregulated compared with the untreated control (* *p* < 0.05, ** *p* < 0.01, *** *p* < 0.001, Student’s *t*-test). (**b**) Correlation analysis of gene expression under drought stress. (**c**) Correlation analysis of gene expression under salt stress. (**d**) Correlation analysis of gene expression under cold stress.

Low temperature, drought, and salinity are important factors affecting maize yield. Screening for genes resistant to abiotic stress can provide genetic resources for breeding resistant maize. In the abiotic stress pathway, some transcription factors are also important components. To investigate the function of maize *GATA* genes in abiotic stress resistance, the expression levels of maize *GATA* genes under low-temperature, drought, and salt stress were analyzed using RT-qPCR, and the correlation analysis of gene expression levels was conducted. The results of low-temperature stress expression levels (Figure 10a) and correlation analysis (Figure 10b) showed that the expression levels of *ZmGATA11* and *ZmGATA18* were significantly lower than the control at 12, 24, 36, and 48 h after low-temperature stress. Their expression levels had a correlation coefficient of 0.86, indicating a negative response to low-temperature stress. On the contrary, the expression levels of *ZmGATA7*, *ZmGATA10*, and *ZmGATA41* were higher than the control, with correlation coefficients of *ZmGATA7/10* = 0.78, *ZmGATA7/41* = 0.98, and *ZmGATA10/41* = 0.88, indicating a significant positive correlation in their expression levels and a possible positive response to low-temperature stress.

The expression levels and correlation analysis of maize *GATA* genes under drought stress are shown in Figure 10a,c. According to the correlation clustering results, *ZmGATA7*, *ZmGATA10*, *ZmGATA19*, *ZmGATA37*, and *ZmGATA41* show strong correlation with each other. Compared to the control, they exhibit the same expression pattern with lower expression levels at 12 h, 24 h, and 48 h and the opposite pattern at 36 h. *ZmGATA18*, on the other hand, has higher expression than the control at 12 h but significantly lower expression at other time points. Based on the expression pattern, it can be observed that the expression pattern of *GATA* genes after drought stress mainly involves an initial increase in expression levels followed by a continuous decrease with a longer treatment duration.

For salt stress, the results in Figure 10a,d show that *ZmGATA10*, *ZmGATA19*, *ZmGATA32*, and *ZmGATA40* have higher expression levels than the control at all four different stress time points. *ZmGATA7* and *ZmGATA27* show upregulation at 12 h and 24 h and downregulation at 36 h and 48 h. The results indicate that there is diversity in the expression patterns of maize *GATA* genes under salt stress, and the expression levels show regularity at different stress time points.

## 3. Discussion

The recent pan-gene family analysis of *bHLH* in barley has provided methods and insights for pan-gene family analysis in other species. Therefore, analyzing the pan-gene family of maize *GATA* is particularly important, as it can provide theoretical support for subsequent analyses [7]. The maize pan-genome comprises 26 high-quality, chromosome-level genomes that include genes absent from the reference genome. Compared to gene family analyses based solely on the reference genome, pan-genomic analysis is more comprehensive and can identify *ZmGATA* genes that are not present in the reference genome. Using maize pan-genomic data, we identified 34 non-reference *ZmGATA* genes in addition to the 41 *ZmGATA* genes present in the reference genome. Notably, even if a gene is present in the reference genome, it may be missing in the genomes of other maize varieties. Among the 75 *ZmGATA* genes, only 58 were found to be conserved across all varieties. *GATA60* and *GATA52* are found only in Ki11; *GATA61* and *GATA64/GATA65* are found only in Ki3 and M37W. These three corn varieties are all of tropical lineage and exhibit good disease resistance; therefore, the unique *GATA* genes present in these varieties may be associated with their disease resistance. For example, an analysis of *TPS* genes in the maize pan-genome revealed that three *ZmTPS* genes were absent from the B73 reference genome [34], while one *OsTPS* gene was missing from the rice reference genome [35]. Despite the quantitative variation in *ZmGATA* gene counts, deletions in some varieties did not lead to a significant reduction in the overall *GATA* gene expression levels. This is likely due to functional redundancy within gene families: when certain genes are missing, other members of the same family can compensate by upregulating their expression to maintain normal plant physiological functions. Such compensatory mechanisms have been observed previously: for instance, when the *CLV1* gene is deleted, its paralogous homologous genes are upregulated to compensate for its role in plant stem cell endocytosis; in *Arabidopsis*, the MADS-box genes *AP1* and *CAL* both contribute to floral organ development. The *ap1* single mutant exhibits only abnormal inflorescences, while the *ap1/cal* double mutant displays infinitely proliferating inflorescences, indicating functional redundancy [36]. In rice, *OsNAC5* works synergistically with *OsNAC6*, and when *OsNAC5* is knocked down, *OsNAC6* expression is upregulated to partially compensate for its function [37]. Notably, *ZmGATA4*, *ZmGATA15*, and *ZmGATA17* were never deleted simultaneously in any variety, as shown in the presence/absence variants (PAVs) Figure 1b. This mutually exclusive deletion pattern suggests that these three genes may form a safeguard mechanism through functional redundancy, ensuring that at least one gene is present in all varieties to maintain normal function.

In this study, we discovered that structural variations (SVs) influence the expression of *ZmGATA8*, which warrants further exploration. Previous research has shown that the promoter region of the maize *bZIP68* gene differs between the ancestral *Z. mays* ssp. *parviglumis* and *Z. mays* ssp. *mexicana*. A 358 bp insertion in the promoter region upregulates *bZIP68* expression in maize, thereby reducing cold tolerance [38]. The effects of SVs on the promoter and downstream regions of *ZmGATA8* are illustrated in Figure 3a. This suggests that structural variations within gene regions may also affect gene expression. For instance, a single-nucleotide polymorphism (SNP) in the promoter region of the maize disease resistance gene *ZmWAKL* significantly increases its expression, enhancing resistance to multiple fungal diseases, such as gray leaf spot [39]. Similarly, natural variations (e.g., insertions/deletions) in the promoter region of the maize plant height gene *ZmTB1* (teosinte branched1) altered its expression during domestication, inhibiting side branch growth and contributing to the upright plant architecture of modern maize [40]. SVs not only influence the expression of *ZmGATA* but also impact gene sequences and structures. These variations may result in the loss of certain functional domains, complicating the identification of gene family members using traditional bioinformatics approaches. Nevertheless, these domain-deleted members may still have critical biological functions. For example, truncated mutants of *ZmNLP8* [41], generated through gene editing (retaining only the RWP-RK domain), still bind to the promoters of target genes and activate the expression of nitrogen-metabolism-related genes, although they lose the ability to interact with PB1. SVs can also lead to structural changes in genes. For example, *ZmGATA8* exhibits variation in the number of untranslated regions (UTRs) across different genomes. Genes with an increased number of UTRs may possess additional functions. Therefore, in this study, we identified several GATA genes in maize with an increased number of untranslated regions, providing valuable resources for a deeper understanding of the role of *GATA* genes in maize.

With the advancement of high-throughput sequencing technologies, omics studies, especially genomics and transcriptomics, have been extensively carried out. Maize transcriptome sequencing data, validated through RT-qPCR analysis, have been proven reliable; thus, effectively utilizing these maize transcriptome data can not only reduce costs but also facilitate deep data mining. This study analyzes the expression patterns of *GATA* genes in different tissue parts and under abiotic stress, using the maize transcriptome data available on the MaizeGDB website through the qTeller module. *GATA* genes in maize exhibit varying levels of expression across different tissue parts and developmental stages, correlating with their functions. In *Arabidopsis*, *GNC* (*AtGATA21*) and *CGA1* (*AtGATA22*) are considered to be broadly involved in the regulation of chlorophyll levels, chloroplast size, photosynthetic efficiency, and carbon–nitrogen metabolism [42]. Similarly, *Os02g12790* (*OsGATA11*) in rice plays a significant role in regulating chlorophyll levels and carbon–nitrogen metabolism [42,43,44]. Homologous genes in maize, *ZmGATA20* and *ZmGATA34*, characterized by higher expression levels in mature leaves and the presence of photoperiod-related elements in their promoters, are likely involved in plant chlorophyll levels and photomorphogenesis. *GATA* also plays a significant role in the regulation of plant photoreceptor pathways, with *AT2G45050* (*AtGATA2*) identified as a key transcriptional regulator integrating brassinosteroid (BR) and photoreceptor signaling pathways [45]. *ZmGATA1* and *ZmGATA35*, homologs of *AtGATA2*, not expressed in the embryo and endosperm but highly expressed in green tissues, may also participate in photoreceptor signaling. Notably, *ZmGATA38* and *ZmGATA39* are specifically expressed in the endosperm, a site for starch synthesis and storage. The regulation of genes related to the starch synthesis metabolic pathway is highly expressed in the endosperm. Reported NAC transcription factors *ZmNAC34* [46], *ZmNAC36* [47], and *ZmNAC126* [48] are highly expressed in the endosperm and significantly associated with the expression of starch-synthesis-related genes, playing roles in starch synthesis. Correlation analysis and expression validation also show that *ZmGATA38* and *ZmGATA39* are significantly related to some starch-synthesis-related genes, with significant expression differences in maize materials with different starch contents, suggesting their potentially important role in maize starch synthesis.

The expression profiles under abiotic stress indicate that several maize *GATA* genes, such as *ZmGATA11*, *ZmGATA28*, *ZmGATA37*, and *ZmGATA41*, are associated with drought, salt, and cold stress, exhibiting downregulation or upregulation. Previous findings have shown that in rice, *OsGATA16* enhances seedling cold tolerance by repressing the expression of *OsWRKY45-1* [49]. In *Arabidopsis*, *AtGNC* (*AtGATA21*) and *AtGNL* (*AtGATA22*) have been found to increase seedling survival rates under cold stress, indicating a link to cold stress resistance [50]. Evolutionary analysis suggests that *ZmGATA20* and *ZmGATA34*, homologous to these genes, also respond to cold stress, as evidenced by their expression profiles. In rice, *OsGATA8* enhances salt tolerance through water balance, light, and efficiency [51], with maize genes *ZmGATA10*, *ZmGATA24*, and *ZmGATA28* being homologous to it and also associated with salt stress. In wheat, *TaGATA62* and *TaGATA73* are related to drought tolerance, and maize expression profiles show that genes such as *ZmGATA37* and *ZmGATA41* are affected by drought stress [52]. Therefore, maize *GATA* genes play significant roles in abiotic stress responses.

## 4. Materials and Methods

### 4.1. Identification of Maize GATA Gene Family

The genomic sequences and annotation data for 26 maize genomes were obtained from the study by Hufford et al. [33]. The 26 maize genomes are classified into six groups based on characteristics and distribution areas: stiff-stalk heterotic (B73); non-stiff-stalk heterotic (B97, Ky21, M162W, Ms71, Oh43, and Oh7B); mixed tropical–temperate ancestry (M37W, Mo18W, Tx303); popcorn (HP301); sweet corn (P39, II14H); and tropical (CML52, CML69, CML103, CML228, CML247, CML277, CML322, CML333, Ki3, Ki11, NC350, NC358, Tzi8). These varieties are primarily from Africa, Asia, and the Americas. The Hidden Markov Model (HMM) configuration file for the GATA domain was retrieved from the Pfam (http://pfam.xfam.org/, accessed on 1 March 2025) database. The GATA domain was identified using HMMER 3.3.2 [53], with an e-value threshold set to e < 1 × 10^−5^. Candidate *GATA* genes were submitted to SMART (http://smart.embl-heidelberg.de/ accessed on 5 March 2025) [54] and the CD-search module of the NCBI to confirm the presence of the GATA domain. Finally, *GATA* genes displaying collinearity across the 26 maize germplasms were classified as *GATA* members.

### 4.2. Phylogenetic Analysis of and Presence/Absence Variation in ZmGATA Gene Family

To classify the maize GATA proteins, we constructed a phylogenetic tree using both *Arabidopsis* and maize GATA proteins. A phylogenetic analysis was performed using GATA protein sequences from both *Arabidopsis* and maize. The *Arabidopsis* GATA protein sequences were obtained from the TAIR10 (https://www.arabidopsis.org/ accessed on 5 March 2025) database. Multiple sequence alignments were conducted using MAFFT v7.490 [55], and the phylogenetic tree was constructed with IQTREE v2.3.6 [56]. The final visualization was generated using TVBOT [57]. The presence/absence variation (PAV) data for GATA were derived from the study by Hufford et al., and the presence or absence of each GATA member across the 26 maize germplasms was illustrated in a heatmap, generated using TBtools II v2.301 [58].

### 4.3. Ka/Ks Calculation

The GATA proteins and coding sequences from the 26 maize genomes were obtained from the study by Hufford et al. [33]. Bulk sequence alignment was performed using ParaAT v2.0 [59], and the Ks/Ks ratios were calculated with the KaKs Calculator [60]. The distribution of Ka/Ks values was visualized as a mountain plot using the R packages ggridges and ggplot2 (v4.0.3) [61]. A heatmap illustrating the proportion of *ZmGATA* genes with Ka/Ks values greater than 1 was generated using the R package ComplexHeatmap [62].

### 4.4. Analysis of Expression of GATAs Overlapped with SVs

The locations of SVs in each maize variety and the gene expression data for the 26 varieties were derived from the study by Hufford et al. [33]. Gene variation annotation was conducted using ANNOVAR v1.0 [63], with the B73 maize genome as the reference. Built-in scripts were employed to determine whether *GATA* genes in B73 overlapped with SVs in other varieties. If a *GATA* gene contained variation sites in a variety, the *GATA* expression data for that variety were treated as the expression data for the SV gene; otherwise, they were considered as expression data for genes without SVs. The Pearson correlation coefficient between the presence of SVs in the gene and the expression levels was calculated. *GATAs* with *p* < 0.05 and |r| > 0.3 were regarded as having significantly altered expression levels due to the presence of SVs. Significant differences between typical and atypical *GATA* genes were assessed using *t*-tests and Wilcoxon tests.

### 4.5. Analysis of Gene Structure of ZmGATAs

The genome annotation file in General Feature Format (GFF) was obtained from https://ftp.gramene.org/maize/v4/gff3/ (accessed on 3 March 2025). The MEME tool was utilized to investigate the motifs present in the ZmGATA protein sequence [64]. The gene encoding the protein of interest exhibits structural variations (SVs) that notably influence the expression levels of the gene. Following this, TBtools II v2.301 was employed to visualize the files generated in the previous analysis in conjunction with the GFF file, facilitating the construction of the gene structure diagram [58].

### 4.6. Collinearity Analysis of GATA Family Genes

The target proteins were aligned against the v4 B73 proteome dataset (https://maizegdb.org/, accessed on 5 March 2025) through BLASTp analysis, resulting in the generation of an m8-format file with a significance cutoff of e < 1×10^−5^. Gene chromosome collinearity was analyzed using the m8-format file and General Feature Format (GFF) file of the v4 B73 genome. The analysis was performed using a collinearity detection toolkit based on the adjusted MCScan method.

### 4.7. Expression Profile Analysis of Maize GATA Genes

The development of maize involves both nutritional and reproductive growth, with the main organs of nutritional growth being the roots, stems, and leaves and the main organs of reproductive growth being the flowers, ears, and seeds. The public database PPRD (Plant Public RNA-seq Database) (http://ipf.sustech.edu.cn/pub/zmrna/, accessed on 5 March 2025) was used to download the expression data (FPKM) of maize B73 in different tissues and under different stress conditions (salt, drought, and cold), and TBtools II was used for the heatmap [58,64]. The specific expression dataset comes from the data PRJNA171684, SRP010680, SRR447831-SRR447847, and SRR447948-SRR447950.

### 4.8. Construction of Co-Expression Network of GATA Genes in Maize, Analysis of Three-Dimensional Structure of Proteins, and Analysis of Protein Interactions

The three-dimensional structure of maize GATA proteins was predicted using the online tool SWISS-MODEL; the obtained PDB format file was analyzed, and the GATA domain was labeled using Pymol v2.0. The protein–protein interactions of maize GATA proteins were predicted using the protein interaction database STRING (https://cn.string-db.org/ accessed on 5 March 2025), and the results were computed and visualized using Cytoscape 3.9.1. A co-expression network of maize *GATA* genes was constructed based on the co-expression network results published on the MaizeNetome (http://minteractome.ncpgr.cn/searchelement.php, accessed on 9 March 2025) website, which is based on the B73 v4 version.

### 4.9. Plant Growth and Treatment

The experimental materials were grown in a greenhouse at the Institute of Agricultural Biotechnology, Jilin Academy of Agricultural Sciences, Changchun, China. In this study, maize variety B73 was used as the material. B73 was the first maize variety to be sequenced and has, therefore, been widely used in various studies. The seeds used in the experiments were obtained from the experimental base of the Jilin Academy of Agricultural Sciences, Gongzhuling, China. Seeds were planted in a mixed substrate (soil–vermiculite = 7:3) under 16 h of light and 8 h of darkness. After the germinated plants had grown five leaves, a salt, drought, and low-temperature stress environment was simulated using 200 mmol/L NaCl, 20% PEG 6000, and 4 °C. The sampling of B73 seedlings was conducted at the V5 (five-leaf) stage. Tissue samples were collected from the fifth fully expanded leaf (counted from the base), which represents metabolically active tissue at this developmental stage. Samples were collected at 12, 24, 36, and 48 h of sample stress treatment and stored at −80 °C for downstream analysis. The grains of maize B73 and its mutant *ae1* were collected at 8, 18, 28, and 38 days after pollination (DAPs) to analyze the impact of maize *GATA* genes on starch synthesis. These samples were frozen in liquid nitrogen and stored at −80 °C.

### 4.10. Quantitative RT-PCR Validation

Real-time fluorescent quantitative PCR was performed using a Step One Plus PCR system (Thermo Fisher Scientific, Boston, MA, USA). The EasyScript^®^ First-Strand cDNA Synthesis Super Mix reagent kit (TransGen Biotech, Beijing, China) was used to synthesize first-strand cDNA, and RT-qPCR was performed using a TransStart^®^ Top Green qPCR Super Mix kit (TransGen Biotech, Beijing, China) with 18S rRNA as a reference gene. The total PCR volume was 20 μL. The experimental procedure consisted of pre-denaturation at 95 °C for 30 s, followed by 40 cycles at 95 °C for 5 s, 58 °C for 15 s, and 72 °C for 10 s. The experiment was repeated three times, and 2^−ΔΔCt^ was used to calculate the relative expression [65]. The gene primer sequences are shown in Table S1.

### 4.11. Determination of Starch Content and Branching Enzyme Activity

This study employed heat-stable α-amylase to hydrolyze starch into a mixture of branched and linear maltodextrins. Following this, glucoamylase was utilized to quantitatively convert the maltodextrins into D-glucose. The starch content was subsequently assessed using a colorimetric method, which involved the reaction of glucose oxidase, peroxidase, and D-glucose, resulting in a measurable color change. The starch content is reported as a percentage, indicating the quantity of starch present in 1 g of corn material. The starch branching enzyme (SBE) plays a crucial role in the production of branched starch by cleaving linear glucan chains and facilitating the formation of branched structures. When amylose interacts with iodine, it forms a complex that displays a distinct absorption peak at 660 nm. The enzymatic activity of the SBE results in a reduction in the amylose content, which in turn decreases the absorbance of the starch–iodine complex at 660 nm. This variation in the absorbance can be utilized to assess the activity of the starch branching enzyme, expressed as a percentage in comparison to a blank control. All reagents utilized in the experiments described above were sourced from Sigma (Merck KGaA, Darmstadt, Germany) and were of analytical quality.

## 5. Conclusions

In conclusion, we performed an in-depth analysis of the *GATA* gene family in the maize pan-genome, revealing significant variation in the number of *GATA* genes across different maize varieties. The pan-genomic analysis identified more *GATA* genes compared to a single genome, and we categorized these genes to distinguish between core and private genes. Structural variations in individual genes at the pan-genomic level had a notable impact on gene expression. *GATA* genes exhibited tissue-specific expression in maize, with genes specifically expressed in the endosperm being suggested to play a role in starch biosynthesis. Further analyses of protein interactions, co-expression networks, and responses to abiotic stresses underscored the crucial role of *GATA* genes in maize under low-temperature, drought, and salt stress. Together, these findings deepen our understanding of the molecular mechanisms underlying maize’s response to environmental stress and offer valuable new targets for improving maize’s tolerance to abiotic stresses.

## Data Availability

The original contributions presented in this study are included in the article/Supplementary Materials; further inquiries can be directed to the corresponding authors.

## Supplementary Materials

The following supporting information can be downloaded at https://www.mdpi.com/article/10.3390/plants14111693/s1, Figure S1: Identification of *GATA* genes in corn reference genome B73; Figure S2: The conserved motifs of GATA proteins in maize varieties B73 and Oh43; FigureS3: Indicators of Starch Synthesis in B73 and AE1. Table S1: Gene primer sequences.
