# Supplementary material for: Pan-Genome-Wide Investigation and Expression Analysis of GATA Gene Family in Maize
Source: Plants (Basel). 2025 Jun 1;14(11):1693. doi: 10.3390/plants14111693 (PMC12158138; doi:10.3390/plants14111693)
Supplement: Supplementary file 1 [file plants-14-01693-s001.zip › FigureS1.pdf]

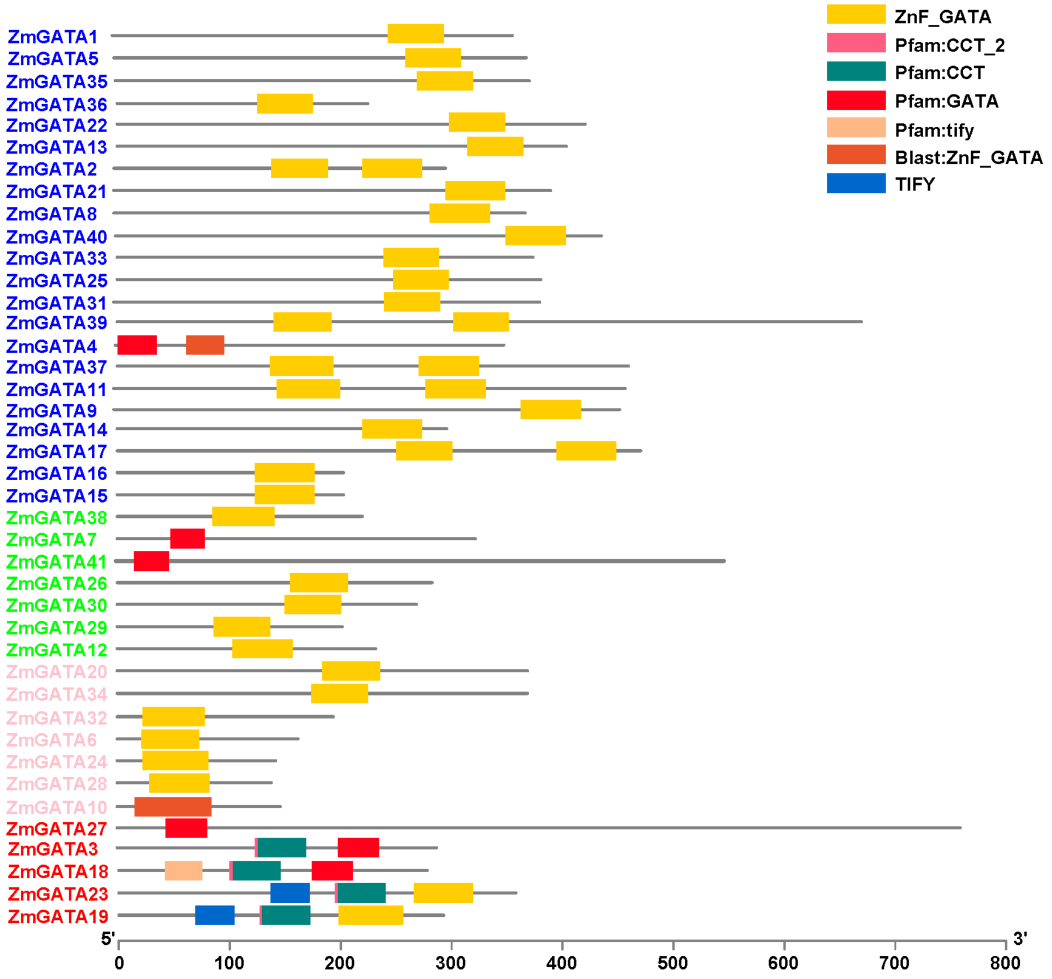

Figure S1. The domain of GATA proteins in maize B73. Each member contains at least one GATA protein domain, but ZmGATA2, ZmGATA39, ZmGATA4, ZmGATA37, ZmGATA11, and ZmGATA17 contain two GATA protein domains.
