# Supplementary material for: Pan-Genome-Wide Investigation and Expression Analysis of GATA Gene Family in Maize
Source: Plants (Basel). 2025 Jun 1;14(11):1693. doi: 10.3390/plants14111693 (PMC12158138; doi:10.3390/plants14111693)
Supplement: Supplementary file 1 [file plants-14-01693-s001.zip › FigureS2.pdf]

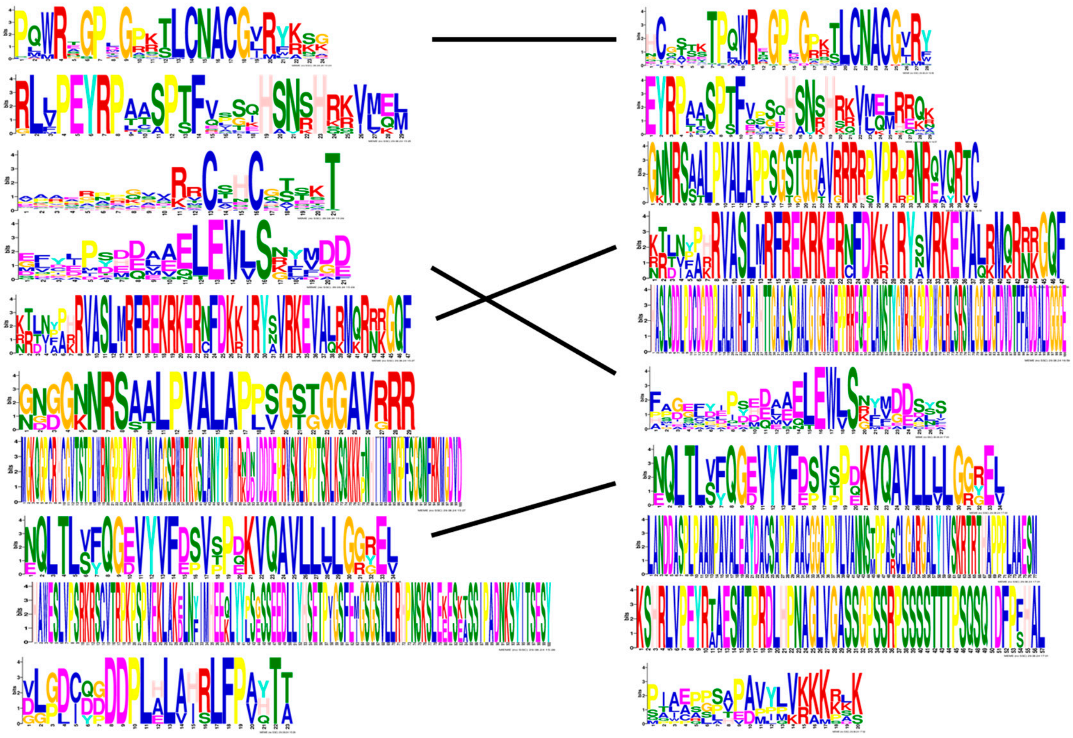

FigureS2. The conserved motifs of GATA proteins in maize varieties B73 and Oh43. In the figure, the left side and right side represent the motifs of B73 and Oh43, respectively. Four motifs are connected by lines, indicating that they are conserved.
