# Supplementary material for: Pan-Genome-Wide Investigation and Expression Analysis of GATA Gene Family in Maize
Source: Plants (Basel). 2025 Jun 1;14(11):1693. doi: 10.3390/plants14111693 (PMC12158138; doi:10.3390/plants14111693)
Supplement: Supplementary file 1 [file plants-14-01693-s001.zip › FigureS3.pdf]

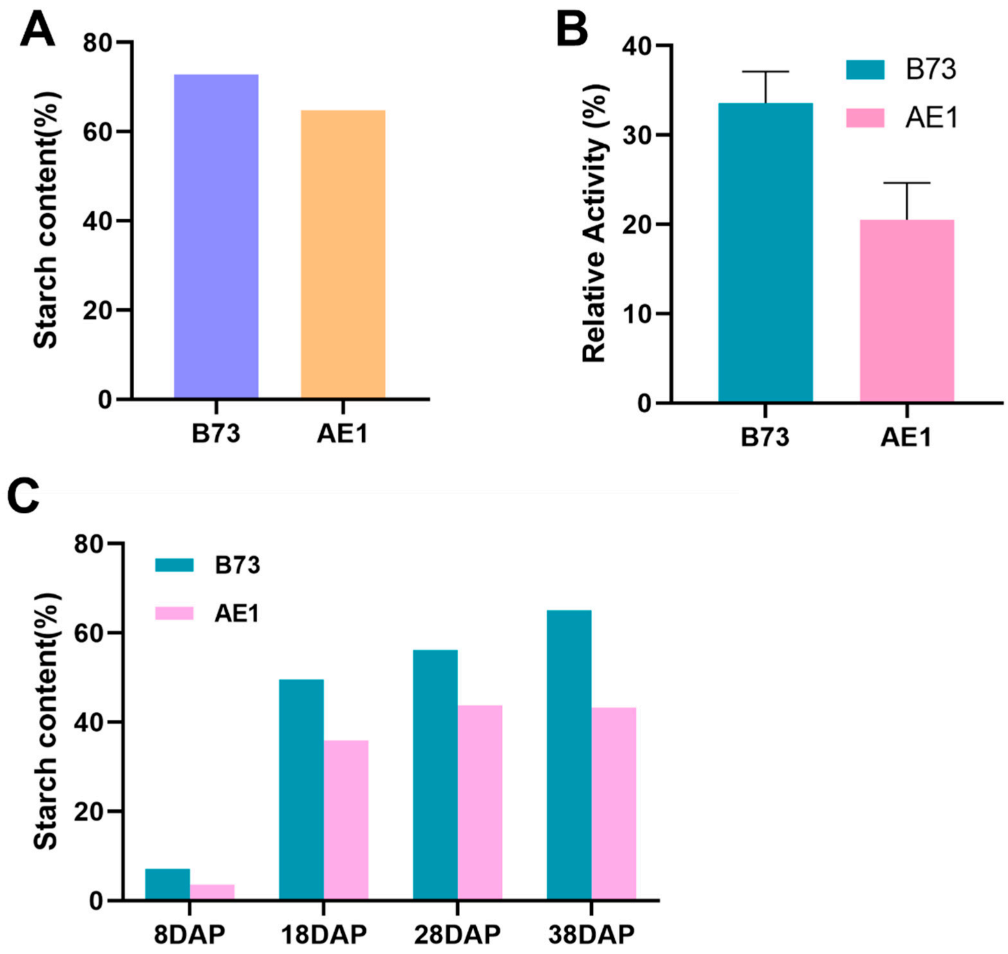

FigureS3. Indicators of Starch Synthesis in B73 and AE1. **(A)** Starch content of mature grains in B73 and AE1. **(B)** Activity of starch branching enzyme in B73 and AE1. **(C)** Starch content of B73 and AE1 grains at different days after pollination(DAP).
